# The structure of the fruit peel in two varieties of *Malus domestica* Borkh. (Rosaceae) before and after storage

**DOI:** 10.1007/s00709-012-0454-y

**Published:** 2012-09-21

**Authors:** Agata Konarska

**Affiliations:** Department of Botany, University of Life Sciences, Akademicka 15, 20-950 Lublin, Poland

**Keywords:** Apple fruit, Epidermis and hypodermis, Cuticle, Epicuticular wax, Microcracks, Water transpiration

## Abstract

The structure of fruit peel of two apple varieties ‘Szampion’ and ‘Jonagold’ was investigated using light microscopy as well as scanning and transmission electron microscopy. The samples were taken immediately after harvest and after 6-month controlled atmosphere storage. The Szampion and Jonagold fruit differed in terms of the surface type, number of lenticels, thickness of the cuticular epithelium, height of epidermal cells and thickness of the hypodermis as well as the amount of crystalline wax and the number of microcracks formed on the fruit surface. The 6-month storage resulted in fruit weight loss, increased numbers and depth of microcracks, thickening of the amorphous wax layer and enhanced production of platelet forms of crystalline wax, which filled the microcracks abundantly. Compared with Jonagold, the Szampion fruit exhibited a fewer lenticels, a bigger number of microcracks, smaller amounts of crystalline wax and more substantial weight loss. The apple varieties studied had a reticulate–lamellate cuticle, and at harvest, the epidermal and hypodermal cells contained numerous amyloplasts filled with starch grains, which were not found after the storage period. Additionally, after storage, the cell protoplasts in the apple peel displayed a disorganised structure, and their vacuoles contained fragments of cell membranes, intravacuolar precipitates and deposits, and spherical bodies. The results may facilitate better understanding of changes occurring in fruits of Szampion and Jonagold during storage and help choose the best storage conditions to reduce loss of weight and prevent impairment of fruit quality.

## Introduction

Apples are the fourth most widely produced fruit in the world. Poland is one of the leading exporters and the biggest apple producer in the European Union. Over the past 5 years, Poland’s apple exports have raised considerably, which is related to, e.g. higher quality of the fruit (Makosz 2011). Consumers appreciate apples that are aromatic, firm-textured, juicy and healthy and have a not too sticky surface (Czernyszewicz 2007). Fruit quality is influenced by many factors: selection of a variety, climate conditions, methods of cultivation, the harvesting season and storage conditions. The quality (attractiveness) of apples is also determined by the peel structure, i.e. the surface layer of the fruit, which is a barrier between its internal and external environments. Fruits are exposed to a variety of unfavourable mechanical, climatic and biological factors while they grow on the tree or during harvest, transport and storage (Jenks et al. 1994; Markstadter et al. 2000). The apple peel is composed of epidermis covered with a cuticle and a multi-layered hypodermis, i.e. a mechanical tissue. The cuticle, i.e. a lipid-type epithelium with a non-uniform structure, plays the most important protective role against the aforementioned factors (Riederer and Schreiber 1995). The internal layer of the cuticle that adheres to the epidermal cell wall forms the so-called cuticular layer, which is most commonly reticulate and contains lipid substances and polysaccharides. The external layer of the cuticle, which borders the external environment, is referred to as cuticle proper; it is formed mainly of lipids and may belong to the reticulate or lamellate type (Holloway 1982; Jeffree 1996; Kerstiens 1996). The surface of the cuticle proper contains a layer of epicuticular waxes in the form of a continuous film of amorphous wax and various forms of crystalline wax (Baker 1982; Jeffree 1986; Barthlott 1990; Barthlott et al. 1998). Epicuticular waxes may contain hydrocarbons, alcohols, aldehydes, fatty acids, diols, esters, B-diketones, terpenoids and phenolic compounds (Walton 1990; Bianchi 1995), and their variable composition determines the wax layer structure (Wettstein-Knowles von 1995; Barthlott et al. 2003). The characteristics and composition of apple cuticular wax change in response to environmental stresses such as rain acidity (Rinallo and Mori 1996), temperature (Lurie et al. 1996; Roy et al. 1996) and radiation (Kasperbauer and Wilkinson 1995). TEM examinations show that cuticle contains polysaccharide microtubules that form a reticulate network through which water transpiration (cuticular transpiration) and transport of epicuticular wax molecules take place (Neinnhuis et al. 2001). Water molecules diffuse through open lenticels located in the fruit peel and via microcracks formed on the apple surface after harvest or during the storage period (Riederer and Schreiber 1995; Maguire et al. 1999; Veraverbeke et al. 2003a). Water loss leads to a decrease in fruit weight and firmness loss (Hatfield and Knee 1988; De Bellie et al. 2000; Veraverbeke et al. 2001a). The amount of transpired water depends on numerous factors, e.g. conditions of fruit growth and ripening (climate, fertilisation, water supply and fruit health), conditions prevailing in the storehouse (temperature, O_2_, CO_2_ and humidity), length of storage and internal factors, i.e. the structure of the peel and, in particular, of the cuticle and epicuticular wax (Riederer and Schreiber 2001; Burghardt and Riederer 2006).

‘Jonagold’ and ‘Szampion’ are widely known important commercial apple varieties grown over large areas in Poland and across Europe. The fruits are highly appreciated by consumers due to their colour, size, aroma and flavour (Kader 1999; Plocharski and Konopacka 1999). They are characterised by long-term storage, although the shelf life varies between the varieties.

The aim of the study was to identify quantitative and qualitative changes in the peel structure in the Szampion and Jonagold apple varieties occurring during 6-month controlled atmosphere storage. To this end, we employed various microscopic techniques. An important aspect of the work was presentation of distortions in the ultrastructure of apple peel cells induced by long-term storage, since literature does not provide sufficient data about this issue. The results obtained may contribute to retaining and increasing the biological value of apples; they will also elucidate the changes in fruits occurring during their development, ripening and storage.

## Materials and methods

Fruits of two winter apple varieties—Szampion and Jonagold—were examined in the years 2008–2010 in two periods: when harvested at the preclimacteric stage (late September/early October) and after 6-month controlled atmosphere (CA) storage (late March/early April) (O_2_ 2 %, CO_2_ 3 %, temperature +3 °C and relative humidity 90–95 %). The fruits came from a private orchard in the Lublin region (Poland) in which conventional growing methods are used. Forty medium-sized, similarly coloured apples that were free of defect were collected from the central part of randomly chosen trees. Special care was taken to avoid touching the fruit surface area intended for observation while picking, transporting and preparing the apple to SEM (to avoid rubbing off and destruction of the wax layer). The individual weight of each with 20 apples was determined at harvest and after completion of storage treatment. Weight loss was calculated as percent weight lost from initial fruit weight at harvest. Additionally, lenticels in the equatorial, calyx and pedicel parts were counted in a 4-cm^2^ area in each of the ten Szampion and Jonagold fruits. Further investigations were carried out using light microscopy and scanning and transmission electron microscopy.

### Light microscopy

In the years 2008–2010, after harvest and storage, hand-cut cross sections from fragments of ten fruits of the Szampion and Jonagold varieties were made, either from the blushed side (the red sun-exposed) and the green-shaded side; next, the samples were embedded in glycerol gelatin and viewed under the Nicon SE 102 light microscope. In each slide, the thickness of the cuticle, the height of the epidermal cells (of the primary and secondary layer), the number of layers of hypodermis and its overall thickness were determined in five places. The structure of various types of lenticels was assessed as well. Further, the samples were stained with Lugol’s iodine in order to detect store starch grains in leucoplasts and with Sudan III (a saturated alcoholic solution of Sudan III) to detect lipophilic substances in the cuticle and lenticels.

Semi-thin transverse sections with 0.7 μm thick were stained with 1 % methylene blue with 1 % azur II in a 1 % aqueous solution of sodium tetraborate. The material was fixed and embedded in synthetic resin with the standard method used in transmission electron microscope. Sections were observed by means of a Nicon Eclipse 90i microscope.

### Scanning electron microscopy

Typical fixation of the material for SEM investigations involves dehydration, which can remove or alter lipids that form the wax coating on the apple surface, and critical point drying can shrink and destroy tissues (Roy et al. 1999). Therefore, a modified and simplified methodology was used in order to prevent destruction of the epicuticular wax. Fragments of fruit with peel (5 × 5 × 2 mm) were sampled from each cultivar immediately after the fruits had been collected from the trees or removed from the storehouse. The samples (freshly cut sections) were wiped with a paper towel, carefully mounted onto stubs, sputter-coated with gold and examined under a TESCAN/VEGA LMU scanning electron microscope at an accelerating voltage of 30 kV.

### Transmission electron microscopy

Small samples (2 × 2 × 2 mm) of Szampion and Jonagold fruits were fixed in 2 % paraformaldehyde and 2.5 % glutaraldehyde buffered at pH 7.4 in 0.1 M cacodylate buffer after harvest and 6-month storage. Fixation was performed at room temperature for 2 h, followed by 12 h at 4 °C. When fixed, the samples were rinsed with 0.1 M cacodylate buffer at 4 °C for 24 h and then treated with 1 % OsO_4_. Subsequently, the samples were transferred to re-distilled water and stained with a 0.5 aqueous solution of uranyl acetate. After passage through increasing concentrations of propylene oxide in ethanol and finally through pure propylene oxide, the samples were embedded for 12 h in Spurr Low Viscosity resin at 70 °C. Ultrathin sections (70 nm thick) were treated with an 8 % solution of uranyl acetate in acetic acid and with lead citrate (Spurr 1969). Images were observed and recorded using the FEI Technai G2 Spirit Bio TWIN transmission electron microscope at an accelerating voltage of 120 kV. Images were captured using a Megaview G2 Olympus Soft Imaging Solutions camera.

### Statistical analyses

Data for each variety was analysed separately as a standard deviation and the correlation coefficient at the 5 % level.

## Results

The fruits of the varieties studied differed in terms of the type of the surface. The Szampion variety had a dry and rough, greenish-yellow or yellow peel covered with intensely red, fuzzy-striped blush and light grey lenticels. The fruits of the Jonagold variety had a smooth, sticky, creamy-yellow surface sometimes covered with a weak, fuzzy-striped carmine blush and greyish lenticels. The total number of lenticels was by 27 % higher in cv. Jonagold than in cv. Szampion (Table 1). Significant differences were also found in the number of lenticels in the calyx, pedicle and the equatorial part of the fruits. In Jonalgold, the number of lenticels in the calyx part was by 82 % bigger than in the pedicel (high correlation), while in the Szampion variety, the difference reached over 50 % (Table 1).

**Table 1:** The average the number of lenticels on the surface of the Szampion and Jonagold fruits

Number of lenticels per 4 cm^−2^	Szampion	Jonagold
Total	19 ± 6.12 a	26 ± 21.02 a
Pedicel part	13 ± 1.82 b	10 ± 3.83 c
Equatorial part	20 ± 5.24 e	14 ± 2.77 e
Calyx part	25 ± 2.97 bd	54 ± 6.61 cd

Lowercase letters (a, b, c, d and e) means that these differences are statistically significant

Additionally, after the 6-month storage, the fruits differed in terms of the amount of transpired water; this process was accompanied by decreased fruit weight and firmness. A single Szampion fruit had lost an average of 5.5 % of its weight, while Jonagold 3.9 % (Table 2).

**Table 2:** The fruit weight loss in Szampion and Jonagold during storage in the years 2008–2010

	Szampion			Jonagold		
	2008	2009	2010	2008	2009	2010
The weight of 20 apples at harvest						
Average in the individual years (in g)	190.7	197.3	183.9	192.5	195.7	188.4
Average of the 3 years (in g)	192.3 ± 7.5			192.2 ± 9.1		
The fruit weight loss						
Average in the individual years (in g)	10.4	9.8	11.5	7.2	7.8	7.4
Average of the 3 years (in g)	10.6 ± 0.99			7.5 ± 1.51		
Average of the 3 years (in %)	5.5 ± 0.68			3.9 ± 0.78		

### Light microscopy

The investigations demonstrated that the epidermal lenticels had reached various developmental stages and exhibited diverse shapes, depth and anatomical structures. Some lenticels were flat, while the others were strongly convex. Ruptured cuticle and epidermis were usually found around the lenticels, and the hypodermal cells of the lenticels contained suberin, cutin and/or tannin compounds, which make this tissue yellow-brown. Most frequently observed were closed lenticels filled with hypodermal cells that contained suberin and cutin (forming the so-called closing cork layer) and/or lenticels that contained phellogen or were filled with cutin. The other types were open lenticels with air spaces and without a closing cork layer (the filling cells had cellulose walls) or with tissue cracks extending to the parenchyma. Phellogen containing lenticels were observed relatively rarely (not shown).

**Fig. 1:**
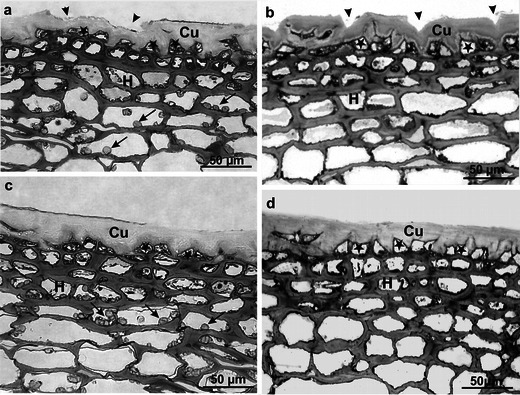
LM. **a–d** Fragments of the cross sections through the fruit peel of Szampion (**a**, **b**) and Jonagold (**c**, **d**) after harvest (**a**, **c**) and after storage (**b**, **d**). Note microcracks (*arrowheads*) in the cuticle (*Cu*) and amyloplasts (*arrows*) in hypodermal cells. Thickened cuticle filling the cell lumen—visible on the anticlinal and internal periclinal epidermal cell walls. *Stars* epidermis, *H* hypodermis

The outer layer, commonly referred to as the peel, was composed of a single- or double-layered epidermis covered by the cuticle and of a multi-layered hypodermis containing tangential or, less frequently, angular collenchyma cells (Fig. 1a–d). Measurements performed under the light microscope showed that the cuticle, staining deep orange with Sudan III, was thicker in both varieties on the blush side than that on the opposite side, both before and after the storage period (Table 3). It was observed that the cuticle over the blush area was by 22 % thicker in Jonagold than in Szampion at the stage of fruit maturity at harvest, and by 10 % after storage. In turn, the cuticle on the shaded side of the Jonagold fruits was by over 17 % thicker than that in the Szampion fruits before storage and by 10 % thicker after that period (Table 3). Moreover, after storage, the cuticle layer in the Szampion variety was by 13.5 % thicker on the blushed side and by 27 % on the shaded side, compared to the period before storage. The differences in the Jonagold variety reached the values of 3 and 16 %, respectively (Table 3).

**Table 3:** The average of the results of measurements of the individual layers forming the apple peel in Szampion and Jonagold in the years 2008–2010

Parameters measured (in μm)	After harvest		After storage	
	Blushing side	Shaded side	Blushing side	Shaded side
Thickness of the cuticle				
Szampion	14.12 ± 1.34 ae	12.28 ± 0.73	16.02 ± 0.91 e	15.64 ± 0.91 b
Jonagold	17.16 ± 0.8 a	14.87 ± 0.75	17.68 ± 1.25	17.19 ± 1.54 b
Thickness of the epidermis				
Szampion—one layer	15.24 ± 1.89 c	15.67 ± 1.54 d	14.8 ± 252	16.05 ± 2.52
Szampion—two layers	32.97 ± 3.22	32.68 ± 3.4	28.81 ± 3.1	31.5 ± 2.69
Jonagold—one layer	17.40 ± 1.88 c	17.40 ± 1.2d g	16.27 ± 2.11	14.70 ± 1.23 g
Jonagold—two layers	31.71 ± 2.33	33.18 ± 2.71	28.73 ± 3.27	27.77 ± 2.5
Thickness of the hypodermis				
Szampion	95.62 ± 9.16	97.93 ± 6.36	98.0 ± 7.42	95.48 ± 4.73
Jonagold	91.33 ± 8.99 f	75.43 ± 4.17 f	96.1 ± 6.95	85.46 ± 7.26
Thickness of the peel				
Szampion	124.98 ± 45.6	125.88 ± 53.23	128.82 ± 48.39	127.1 ± 50.83
The average of the peel	125.40 ± 48.55		128.00 ± 49.21	
Jonagold	125.89 ± 50.34	107.70 ± 52.57	130.05 ± 49.45	117.35 ± 51.73
The average of the peel	116.80 ± 51.39		123.70 ± 50.47	

Lowercase letters (a, b, c, d, e, f, and g) means that these differences are statistically significant

It was found that the cuticle was often produced not only on the external wall of the epidermal cells but also within the anticlinal walls in this tissue, thus producing a relatively thick layer (Fig. 1a–d). Furthermore, primarily after the storage period, additional cuticle deposition was detected on the internal wall of the tangential epidermis, thus the cell lumen was often filled with the cuticle (Fig. 1b, d). The cuticle layer being in contact with the external environment exhibited cracks and fissures of varied width and depth, which did not extend to the epidermal cell walls. This was largely visible in the Szampion variety (Fig. 1a, b).

The single- or double-layered epidermis was composed of small viable cells with a small lumen. During the harvest period, cell divisions were observed along the anticlinal walls of this tissue, which resulted in arrangement of the epidermal cells in pairs (Fig. 1a–d). In the Szampion fruits, the height of cells in the primary layer of the epidermis was similar on both the blushed and shaded part of the fruit after harvest. After the storage period, the shaded part of the fruits exhibited a slight increase (by 2 %) in the height of the epidermal cells, whereas the cell height on the blushed side decreased by 3 % (Table 3). During the harvest period, the height of the external epidermis cells was similar on both sides of the Jonagold fruits; after storage, it decreased by 7 % on the blushed side part and over 18 % on the shaded side of the fruit. After the storage period, the height of epidermal cells in the Szampion variety was by over 8 % lower on the blushed side of the apple than on the shaded side, whereas a reverse relationship was observed in the Jonagold variety, in which the height of the epidermal cells on the blushed side was by 11 % higher than that on the shaded side (Table 3). During the harvest period, the cells of the secondary epidermis layer in the Szampion fruits were slightly more than the cells in the primary layer, but during the storage period, these cells were fewer. In the Jonagold variety, the cells had slightly shorter height than the cells of the primary layer, both before and after storage (Table 3).

The fruit hypodermis was composed of several (three to six) layers of tangential (and less frequently angular) collenchyma, whose flattened tetragonal to hexagonal cells were filled with numerous leucoplasts containing starch grain during the harvest. No such structures were observed after the storage period. The closer distance to the fruit interior, the larger the diameter of the hypodermal cells (Fig. 1a–d). The hypodermis cells in the Szampion variety were characterised by a smaller diameter than those in the collenchyma in the Jonagold variety. The collenchyma layer was thicker in Szampion than in Jonagold in all the variants under study. The greatest difference (30 %) in the thickness of this layer between the varieties was found on the shaded side of the fruit before storage, and the smallest (2 %) on the blushed side after storage (Table 3). In the Szampion variety, the thickness of collenchymas on the blushed side increased during storage by 3 %, but it was similarly reduced on the shaded side. Simultaneously, during the harvest period, the hypodermis layer was by over 2 % thicker on the shaded side of the fruit; after storage, this layer was thicker on the blushed side by almost 3 %. In the case of the Jonagold variety, the collenchyma layer was thicker on the side with the blush than on the shaded side of the fruit by 21 and 13 % before and after the storage, respectively. During the storage period, the thickness of the hypodermis increased by over 5 % on the side with the blush and by over 13 % on the opposite side (Table 3). The total thickness of the peel was greater in the Szampion variety than in Jonagold by 7 % at the harvest time and 3 % after the storage. During storage, each variety displayed an insignificant increase in the peel thickness by 1 and 6 % in Szampion and Jonagold, respectively (Table 3).

### Scanning electron microscopy

**Fig. 2:**
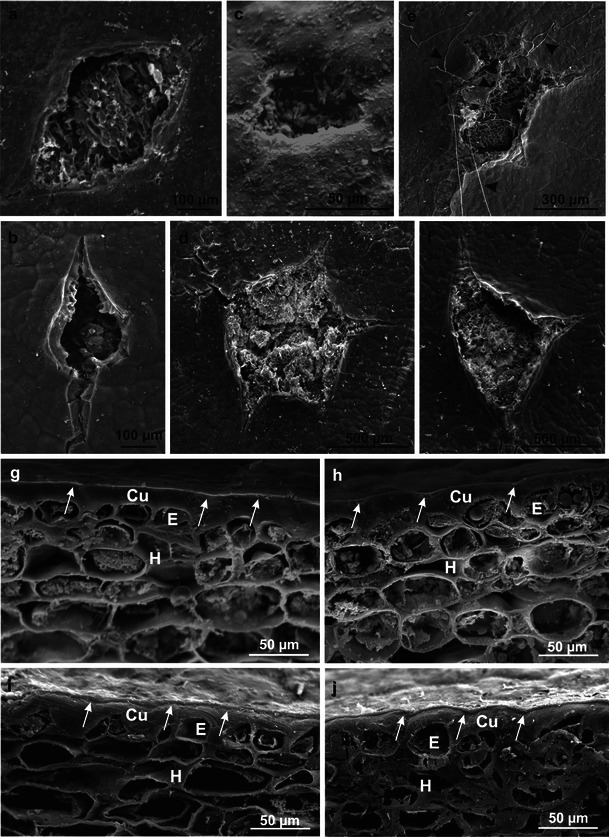
SEM. **a–f** Different types of lenticels on the apple surface: **a**, **e**, **f** Szampion; **b**, **d** Jonagold. Note the mycelial hyphae (*arrowheads*). **g**, **h** Fragments of the cross sections through the fruit peel of Szampion (**g**) and Jonagold (**h**) after harvest. **i**, **j** Fragments of the cross sections through the fruit peel of Szampion (**i**) and Jonagold (**j**) after storage. *Arrows* an amorphous wax film on the cuticular layer, *Cu* cuticle, *E* epidermis, *H* hypodermis

SEM imaging revealed that the fruit epidermis was composed of tetragonal to heptagonal tightly adhering cells. Various shapes of lenticels (oval, star-shaped, lens-shaped and stomata-like) at various developmental stages were visible in the epidermis (Fig. 2a–f). Mycelial hyphae were observed in some lenticels in the Szampion fruits (Fig. 2e).

During the harvest period, the fruit epidermis in both varieties was covered by cuticle, which exhibited an external light and ca. 0.7-μm-thick layer of amorphous wax (Fig. 2g, h) visible in cross sections of the surface fruit tissues. After the storage period, the thickness of the wax layer increased and reached 2.0–2.4 μm and formed a tight, continuous film covering the cuticle surface (Fig. 2i, j).

**Fig. 3:**
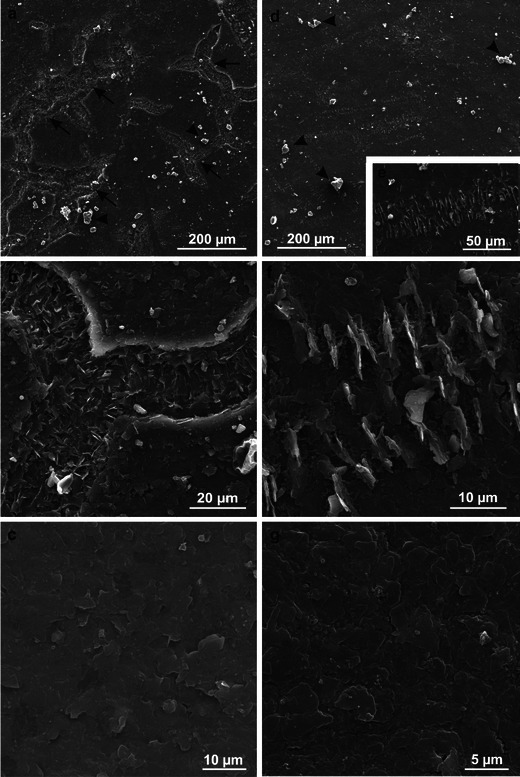
SEM. **a–c** Fragments of the epidermis surface of Szampion fruit after harvest. Note numerous microcracks (*arrows*) and horizontal wax platelets between the microcracks (**c**). **d–g** Fragments of the epidermis surface of Jonagold fruit after harvest. Note vertical wax platelets (**d–f**) and horizontal wax platelets (**g**). *Arrowheads* granular epicuticular wax

Numerous 20–60-μm-wide microcracks were observed on the fruit surface after the harvest, particularly in the Szampion variety (Fig. 3a, b). Crystalline wax, which was more abundant in the Jonagold variety, had the form of platelets of various sizes (max 6.25 × 3.75 μm) and orientation or of different-size granules (lumps) (Fig. 3c–g). The platelets were arranged perpendicularly to the fruit surface thereby forming parallel rows, or they formed a horizontal layer without specific arrangement on the fruit surface. Compared to the Szampion variety, Jonagold exhibited more vertically oriented wax platelets, which were present mainly on the blushed fruit surface.

**Fig. 4:**
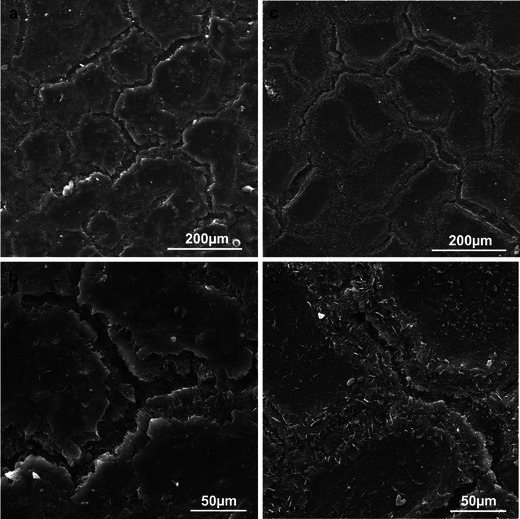
SEM. **a**, **b** Fragments of the epidermis surface of Szampion fruit with microcracks and without vertical platelets of crystalline wax after storage. **c**, **d** Fragments of the epidermis surface of Jonagold fruit with microcracks and vertical and horizontal platelets of crystalline wax after storage

**Fig. 5:**
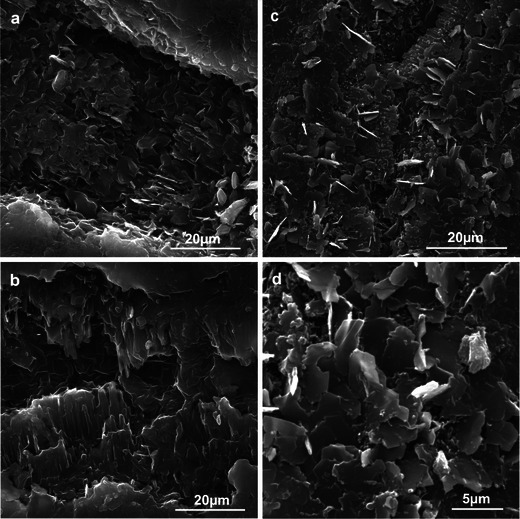
SEM. **a**, **b** Microcracks without vertical platelets of the epicuticular wax on the surface of Szampion fruit after storage. **c**, **d** Microcracks with vertical platelets of the epicuticular wax on the surface of Jonagold fruit after storage

After the 6-month CA storage of the fruit, the number of the microcracks on the apple surface, which were at different developmental stages, increased in both varieties (more abundantly in the Jonagold variety). The width of the microcracks was similar to that observed after the harvest, whereas their depth was greater. They were arranged in various directions along the epidermal cell walls, forming a reticulate network (Fig. 4a–d). The microcracks showed deeper cuticle layers, which were stretched and eventually ruptured (Fig. 5a, b). The crystalline wax on the Szampion fruits assumed a form of horizontal platelets on the cuticle surface; few were vertically oriented inside the microcracks (Fig. 5a, b). In turn, in the Jonagold variety, vertically arranged wax platelets dominated; they were clustered at the edges of the microcracks or filled them (Fig. 5c, d). The wax structures were higher and narrower than those that were found in the freshly harvested fruits. The microcracks were concentrically arranged around the lenticels. Mycelial hyphae were sporadically observed inside the lenticels and microcracks in the Szampion variety.

### Transmission electron microscopy

**Fig. 6:**
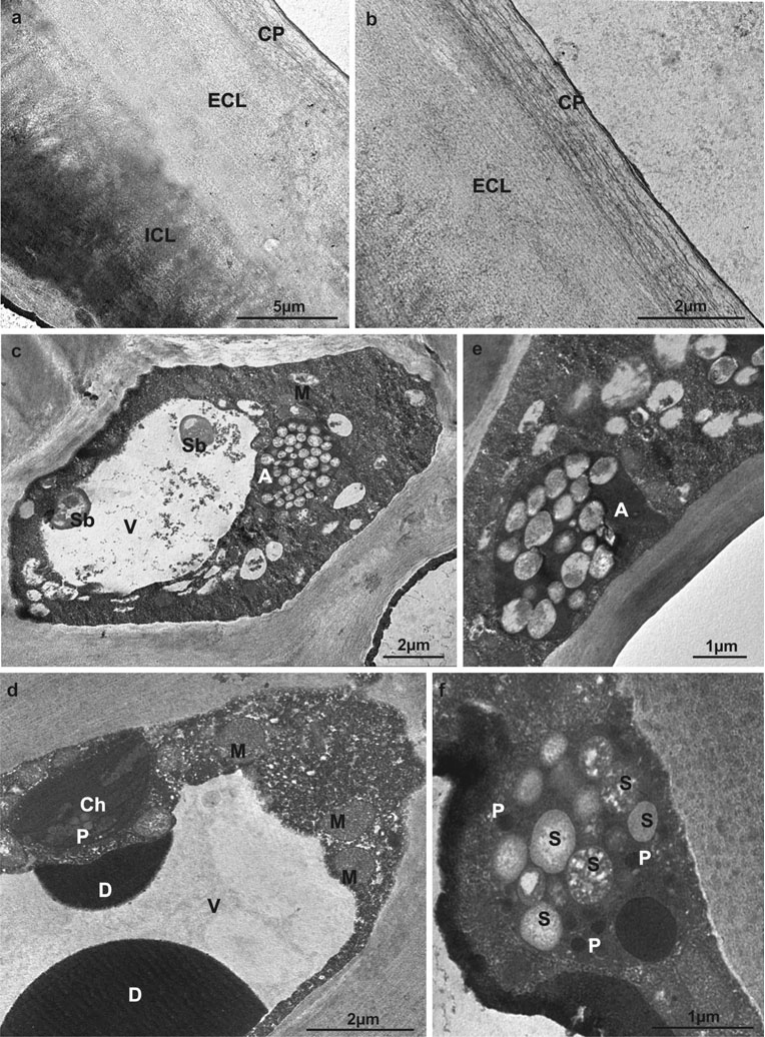
TEM. **a**, **b** Ultrastructure of the Szampion (**a**) and Jonagold (**b**) fruit cuticle after harvest. **c** Epidermal cell of Szampion fruit after harvest. Note the intravacuolar precipitates and spherical bodies (*Sb*). **d** Fragments of the hypodermal cell of Jonagold fruit with chloroplast (*Ch*) and intravacuolar deposits (*D*) after harvest. **e**, **f** Amyloplasts (*A*) with starch grains (*S*) in the hypodermal cells of Szampion (**e**) and Jonagold (**f**) fruit after harvest; *CP* cuticle proper, *ECL* external cuticular layer, *ICL* internal cuticular layer, *V* vacuole, *M* mitochondrion, *P* plastoglobules

The cuticle covering the epidermis of the Szampion and Jonagold apples before and after storage exhibited a non-uniform structure: the several-fold wider internal CL bordering the epidermal cell walls was reticulate, while the thinner (ca. 0.8–1.0 μm) upper CP layer was formed by parallel lamellae (lamellate type). Within the CL, a darker internal CL bordering the epidermal cell walls and a less intensely stained external CL merging into CP were identified (Fig. 6a, b).

During the harvest period, the different-size mature epidermal cells contained one large vacuole or many small vacuoles. The cells were characterised by abundant, dense cytoplasm containing numerous cellular structures (Fig. 6c). The epidermal and hypodermal vacuoles of the Jonagold variety contained spherical bodies and dark spherical deposits of different sizes as well as flocculent, fibrous precipitate (intravacuolar precipitate) (Fig. 6c, d). Chloroplasts containing assimilation starch grains and plastoglobules (Fig. 6d) as well as oval or elongated mitochondria and leucoplasts filled with store starch grains were visible (Fig. 6c–f).

**Fig. 7:**
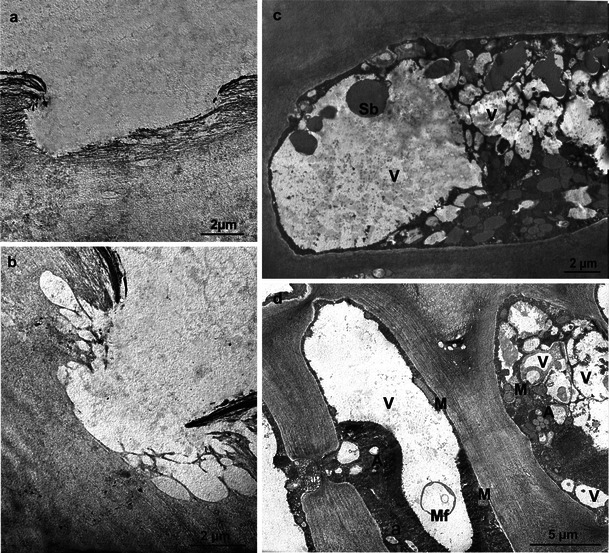
TEM. **a, b** Fragments of the Szampion (**a**) and Jonagold (**b**) fruit cuticles with microcracks after storage. **c**, **d** Fragments of the epidermal cells of Jonagold (**c**) and Szampion (**d**) fruit after storage; *V* vacuoles, *M* mitochondrion, *A* amyloplasts, *Sb* spherical bodies, *Mf* myelin figure

After the 6-month CA storage, in addition to its increased thickness, the cuticle of the apple varieties investigated exhibited a structure similar to that described in the harvest period. However, ruptures that damaged the cuticle proper and deeper parts of the cuticular layer were visible (Fig. 7a, b). The live epidermal cells displayed a distorted structure, where the cytoplasm was divided into compartments containing small vacuoles filled with spherical bodies, myelin figures and probably fragments of membranes (Fig. 7c, d). Cells with typical organisation had parietally located cytoplasm with mitochondria and few leucoplasts containing starch grains (Fig. 7d).

## Discussion

Organoleptic tests revealed significant differences in the type of fruit surface of the varieties examined. The peel in the Szampion apples was rough and dry, whereas in Jonagold it was smooth and greasy. This difference was accompanied by different thickness of the fruit cuticle and epidermis, different amount of water loss during storage (expressed as a decrease in fruit weight), a different area covered by microcracks and different amounts of epicuticular wax produced on the fruit surface. Additionally, the fruits differed in the number of lenticels.

### Thickness of the cuticle and epidermis

The LM measurements showed that storage increased the cuticle thickness in the two varieties, on both the blushed surface and shaded side of the apples. Similar observations of varying thickness of the cuticular epithelium in various parts of fruits have been reported by other authors (Skene 1963; Hull et al. 1975; Veraverbeke et al. 2001b, 2003a). Besides the genetic background, other factors, e.g. climatic and storage conditions, fruit health and microenvironment conditions, affect cuticle thickness (Babos et al. 1984; Glenn et al. 1990; Curry 2001; Homutová and Blažek 2006; Ghafir et al. 2009). Riederer and Schreiber (1995) and Knoche et al. (2000) reported lack of correlations between the thickness of the cuticle and cuticular water permeability. Yet, a thicker cuticle layer on the blushed surface may ensure better protection for the fruit interior against the harmful effects of UV radiation (Solovchenko and Merzlyak 2003).

### Weight losses and lenticels

During the 6-month CA storage, the fruit weight decreased by 5.5 % in Szampion and almost by 4 % in Jonagold, which in each 1,000 tonnes of fruit would cause a 0.5-ton loss. These observations are consistent with the results obtained by Link et al. (2004), who estimate that apples lose from 0.6 to 1.1 % of their weight, during each month of storage, depending on the variety. Hatfield and Knee (1988) have found that a 3–6 % of fruit weight loss during storage may result in withering and hence reduction of apple attractiveness. Such fruits not only lose the firm texture but also the aroma and flavour (Fellman et al. 2003; Scalzo et al. 2003). The long-term storage is likely to have led to such a significant reduction in the weight of both the varieties studied, although location of cultivation and climatic conditions may also be important. The influence of these factors on the amount of water loss has been reported by Veraverbeke et al. (2003b), Müller (2005), as well as Markuszewski and Kopytowski (2008). The results concerning the number of lenticels on the fruit varieties examined are insufficient to infer the effect of lenticels on the water transpiration level, since the number of open and closed lenticels was not assessed. Although the total number of lenticels in the Szampion variety was lower than that in Jonagold, Szampion fruits lost more water during storage. According to Varaverbeke et al. (2003a), Jonagold has 35–42 % of open lenticels. As shown by Maguire et al. (1999), transpiration via lenticels may account for 20 % of water loss, while the rest of water loss proceeds through cuticular transpiration. A different view has been put forward by Veraverbeke et al. (2003a), who have demonstrated that open lenticels mostly influence the global moisture loss, while the effect of cracks and cuticular transpiration is much smaller.

### Microcracks

Already after the harvest in the Szampion variety and only after the storage in Jonagold, the fruit surface exhibited numerous microcracks. Maguire et al. (2000) claim that microcracks are formed during development and growth of the fruit on the tree as well as during storage. In turn, Roy et al. (1999) suggest that no new microcracks appear during storage, but the microcracks formed during fruit growth on the tree are deepened, which according to Knoche and Grimm (2008) takes place when the fruit surface is exposed to water or high humidity. The author of the present paper has observed formation of new microcracks during storage, especially on the Jonagold fruits, which did not exhibit any cuticle microcracks after harvest. The literature suggests that microcracks enhance cuticular transpiration and may cover 40 % of the fruit surface in varieties with a rough peel, thereby increasing the permeability of the cuticle even 15-fold (Maguire et al. 1999). Veraverbeke et al. (2003a) have found that microcracks can occupy a fourfold smaller surface in fruits with a smooth (Jonagold) or rough (‘Elstar’) peel.

The microcracks on the surface in the fruits tested had varying depth and width, but they did not extend to the epidermal cell walls. Similar data on the size of cracks were provided by Glenn et al. (1990), Maguire et al. (1999) and Curry (2009). Roy et al. (1999) has reported that the increasing depth of cracks results from splitting of platelets that form successive wax layers. In turn, Glenn and Poovaiah (1985) argue that cuticular microcracks are formed during fruit growth in the process of degradation of cell wall components and disintegration of walls caused by large amounts of water penetrating this element of the apoplast. This results in a collapse of the wall support structure and formation of microcracks.

### Epicuticular wax

At harvest and after the storage period, differences were found in the amount and structure of epicuticular wax (both amorphous and crystalline) on the surface of the cuticle proper. According to many authors, the presence of epicuticular wax exerts a significant effect on transpiration intensity (Faust and Shear 1972; Babos et al. 1984; Roy et al. 1994; Rinallo and Mori 1996; Belding et al. 1998; Veraverbeke et al. 2001a, 2003b). In their studies on various apple varieties, the authors observed differences in the amount and form of epicuticular wax produced on the fruit surface during ripening, storage and shelf life. The abundance and structure of epicuticular wax is not only largely determined by environmental conditions, e.g. humidity or light intensity, but also by genetic predispositions of the variety (Faust and Shear 1972; Walton 1990; Curry 2009), type of the rootstock, fungicides and surfactants applied (Wolter et al. 1988), and temperature and oxygen concentration during storage (Brackmann and Bangerth 1993).

After the storage period, the thickness of the amorphous wax layer was similar in both fruit varieties. The layer, however, was threefold thicker than that observed at harvest. Crystalline wax was more abundant on the Jonagold fruits after both the harvest and storage periods. A similar thickness of the amorphous wax layer in Jonagold fruit stored for 4 months in CA storehouses was observed by Veraverbeke et al. (2001b). Jeffree (1996) and Koch et al. (2006) suggest that the continuous film of amorphous wax on the fruit surface is the source and site of crystallite synthesis. Apparently, the conditions for synthesis of crystalline wax forms were similar for both the varieties studied. It is probable that in the case of the Szampion variety, a genetic factor prevented synthesis of bigger amounts of crystalline wax forms. Similarly, Faust and Shear (1972) and Roy et al. (1999) have shown that varieties with a rough peel are covered with amorphous wax only and numerous cracks are formed on their surface during fruit growth. In varieties whose surface is additionally covered by crystalline wax, fewer cracks are formed and fruits lose less water during storage, as the crystalline wax inhibits transpiration, whereas amorphous wax does not prevent the process.

The crystalline wax in both the apple tree varieties exhibited varied sizes and orientation. In Jonagold, the wax platelets were arranged perpendicularly to the fruit surface and they filled the microcracks; in the Szampion variety, they formed a flat surface and were sparse at the microcrack site. The platelet form of wax on fruits of various apple varieties was also observed by other authors (Skene 1963; Glenn et al. 1990; Roy et al. 1994; Belding et al. 1998). Koch et al. (2004) suggest that the arrangement of wax crystals (wax platelets) is not accidental. They are usually arranged perpendicularly to the surface of cracks and lenticels in order to protect the fruit against water loss. Curry (2009) argues that the wax microtubules within the crack are distally elongated and form flat aggregates of microcrystalline wax platelets, which undergo polymerization and form an insoluble, semi-permeable cutin matrix (amorphous wax). As the fruit grows, this layer is stretched and flattened, hence, the wax microtubules are torn; this is followed by elongation of their distal fragments, which results in stitching (filling, repairing) of the microcrack. Elongation of microtubules is determined by temperature and humidity of the environment; therefore, the mechanism described (‘Tear and Repair’ or ‘Rip and Stitch’) contributes to reduction of transpiration from fruits growing on the tree at high ambient temperature and low humidity and from fruits stored at high humidity and markedly lowered temperature (Curry 2001, 2009; Müller 2005). According to many authors, synthesis of cuticular waxes proceeds throughout fruit life and continues in the storehouse until the substrate in the epidermal cells is exhausted or necrosis of the tissue takes place (Morice and Shorland 1973; Belding et al. 1998; Curry 2001). In the Szampion variety, where no Tear and Repair mechanism was observed, the fruits were exposed not only to enhanced transpiration but also pathogen infections (both on the tree and in the storehouse), which was indicated by the presence of mycelial hyphae in the microcracks and lenticels. It was observed that more wax platelets were formed on the blushed side of the fruit, which had been exposed to intense solar radiation, than on the shaded side. According to Solovchenko and Merzyak (2003), the presence of crystalline waxes protects cells not only against water loss but also from the harmful effect of UV radiation by increasing reflection and dispersion of sunrays.

### Ultrastructure of the peel

The reticulate–lamellate structure of the cuticle demonstrated by TEM in Szampion and Jonagold fruits corresponds to one of the cuticle types typical of apple trees described by Holloway (1982) and Kerstiens (1996). The reticulate part contains a reticulate network of tubules composed of polysaccharide microfibrilles (Jeffree 1996) through which water and epicuticular wax components are transported onto the cuticle surface (Miller 1982; Riederer and Schreiber 1995; Neinhuis and Barthlott 1997). The cuticle in both the apple varieties exhibited different colouring of the internal and external CL. Similar differences in the colouration of these two layers were also observed by Schmidt and Schönherr (1982), who found that the layers differed in density and chemical composition. Kerstiens (1994) suggests that the lamellate structure of the CP limits transpiration, since the reticulate structure of the cuticle is most permeable, whereas all other cuticle types are characterised by lower permeability.

After the 6-month storage of Szampion and Jonagold fruits, the epidermal and hypodermal cells contained only few starch grains, which was probably connected with previous degradation of starch and its transition to soluble sugars: fructose and sucrose, a process which coincides with fruit maturity for consumption. Such processes occurring in apple peel cells were reported by Berűter (1985) and Brookfield et al. (1997). Additionally, disturbances in the organisation of protoplasts were observed in epidermal and hypodermal cells, while myelin structures, deposits of unknown origin and plasma membrane fragments were detected in the vacuoles. The changes described may indicate a far-reaching process of cell destruction and advanced processes of cell death. Oval, dark deposits observed at harvest in the vacuoles of the peel cells in Jonagold are probably condensed tannins, whose presence in the peel of ripening fruit of other apple varieties was reported by Garry et al. (1995). The extensive literature on the structure of apples, their ripening and storage provides little information on the disorders of the ultrastructure of peel cells caused by long-term storage. Starch grain-filled amyloplasts and the first symptoms of an ultrastructural degeneration was observed (Peng and Zhang 2000) in mature ‘Red Fuji’ fruits. The insufficiency of data on this topic may be related to difficulties in obtaining ultrathin sections through the surface layer of stored fruit as these tissues are non-uniform (different hardness of the cell walls, cell shrinkage, the progressive process of dissolution of the middle lamella and cuticle detachment).

## Abbreviations

LM: Light microscopy
SEM: Scanning electron microscopy
TEM: Transmission electron microscopy
CA: Controlled atmosphere
CL: Cuticular layer
CP: Cuticle proper
